# Role of Nutritional Status in the Treatment Outcome for Esophageal Squamous Cell Carcinoma

**DOI:** 10.3390/nu13092997

**Published:** 2021-08-28

**Authors:** Miao-Fen Chen, Ching-Chuan Hsieh, Ping-Tsung Chen, Ming-Shian Lu

**Affiliations:** 1 Department of Radiation Oncology, Chang Gung Memorial Hospital, Chiayi 61363, Taiwan; 2 College of Medicine, Chang Gung University, Taoyuan 33302, Taiwan; hsiehcgmh@gmail.com; 3 Department of General Surgery, Chang Gung Memorial Hospital, Chiayi 61363, Taiwan; 4 Department of Medical Oncology, Chang Gung Memorial Hospital, Chiayi 61363, Taiwan; chencgmh@gmail.com; 5 Department of Thoracic & Cardiovascular Surgery, Chang Gung Memorial Hospital, Chiayi 61363, Taiwan; lucgmh7@gmail.com

**Keywords:** esophageal cancer, nutrition, immune, prognosis, surgery

## Abstract

Undernourishment is reported to impair treatment response, further leading to poor prognosis for cancer patients. We aimed to investigate the role of nutritional status on the prognosis of squamous cell carcinoma (SCC) of the esophagus, and its correlation with anticancer immune responsiveness. We retrospectively reviewed 340 esophageal-SCC patients who completed curative treatment and received a nutrition evaluation by the Patient-Generated Subjective Global Assessment (PGSGA) score at the beginning and completion of neoadjuvant treatment at our hospital. The correlation between the nutritional status and various clinicopathological parameters and prognosis were examined. In addition, the role of nutritional status in the regulation of the anticancer immune response was also assessed in cancer patients and in a 4-nitroquinoline 1-oxide (4NQO)-induced esophageal tumor model. Our data revealed that malnutrition (patients with a high PGSGA score) was associated with advanced stage and reduced survival rate. Patients in the group with a high PGSGA score were correlated with the higher neutrophil-to-lymphocyte ratio, higher proportion of myeloid-derived-suppressor cells (MDSC) and increased IL-6 level. Furthermore, surgical resection brought the survival benefit to patients in the low PGSGA group, but not for the malnourished patients after neoadjuvant treatment. Using a 4NQO-induced tumor model, we found that nutrition supplementation decreased the rate of invasive tumor formation and attenuated the immune-suppressive microenvironment. In conclusion, malnutrition was associated with poor prognosis in esophageal-SCC patients. Nutritional status evaluated by PGSGA may be useful to guide treatment decisions in clinical practice. Nutritional supplementation is suggested to improve prognosis, and it might be related to augmented anticancer immune response.

## 1. Introduction

Malnutrition is a common problem in patients with aerodigestive tract malignancies [1,2]. Among aerodigestive tract malignancies, esophageal cancer is an aggressive malignancy with high recurrence rate and poor rate of survival [3]. Esophageal cancers exist in two distinct histological types, and squamous cell carcinoma (SCC) is the predominant histologic subtype for esophageal cancer in our country [4]. The treatment outcome depends on patient characteristics, tumor status, and response to treatment [5]. It has been reported that undernourishment can impact tumor progression and survival in cancer patients [6]. The positive effects of nutrition on outcome have been pointed out in patients undergoing chemotherapy/radiotherapy or major elective surgery [7,8]. Considering nutritional status is associated with prognosis, nutritional assessment should be integrated with the multimodal anticancer treatment [9]. Accordingly, we evaluated the nutritional status of esophageal-SCC patients by the Patient-Generated Subjective Global Assessment (PGSGA), and examined its role in the treatment response and prognosis.

Host inflammatory response is recognized as a key regulatory factor in tumor development and progression [10]. Myeloid-derived-suppressor cells (MDSCs), several inflammatory markers, and the neutrophil-to-lymphocyte ratio (NLR) have been reported to induce an immunosuppressive tumor microenvironment and act as prognostic indicators for cancer patients [11,12,13,14]. We previously reported that the NLR was relevant to IL-6 and MDSC levels, and that it was associated with poor prognosis in esophageal-SCC patients [15,16]. Tumor-induced inflammation such as IL-6 has been reported to play a critical role in nutrition metabolism and enhanced body-weight loss in cancer patients [17,18]. Nutritional supplements have been reported to improve the immune response in malnourished patients [19]. Therefore, for cancer treatment, nutritional problems have become an issue that may have a prognostic role in cancer patients and enhance the immune response against cancer [7,20]. In the present study, the relationship between nutritional status and immunologic factors in esophageal-SCC patients was also examined.

## 2. Materials and Methods

### 2.1. Study Population

This study was approved by the Institutional Review Board of Chang Gung Memorial hospital (No. 202001865B0). A total of 340 esophageal-SCC patients who were with stage T2–T4 or regional lymph node involvement and completed curative treatment were enrolled in the study. The curative treatment for esophageal cancer included neoadjuvant concurrent chemoradiotherapy (CCRT) combined with surgery (Surgery group) or definitive CCRT (CCRT group). After neoadjuvant CCRT, surgery was considered for all patients with resectable esophageal cancer. If surgery was contraindicated or the patients refused to undergo surgery, a CCRT boost was given as definitive CCRT.A radiotherapy dose of 45–50.4 Gy combined with 2 courses of chemotherapy were administered for neoadjuvant CCRT. Upon completion of neoadjuvant CCRT, patients underwent systemic workup to determine the treatment response. All esophageal-SCC patients were divided into two groups according to the pretreatment NLR: the high (NLR ≥ 3) and low (NLR < 3) groups. Among these enrolled patients, 253 patients had available immunohistochemistry (IHC) data for IL-6 staining, and 124 had the data of MDSCs labeled as CD11b + CD33+ HLA-DR- and IL-6 levels from the peripheral blood as described previously [15].

### 2.2. Nutritional Assessment

The PGSGA score is a modification of the Subjective Global Assessment (SGA) [21]. The PGSGA has been employed to evaluate the nutritional status of cancer patients [22,23]. The calculation of the PGSGA score involves patient medical records, including weight loss, nutrition impact symptoms, intake and functional capacity. A higher score reflected a greater risk of malnutrition. The results of the PGSGA score fell into grade A (score 0–3) with normal nutrition; grade B (score 4–8) with moderate malnutrition; and grade C (score ≥ 9) with severe malnutrition [24]. In the present study, the nutritional assessment using the PGSGA score was conducted by an experienced dietitian. All enrolled patients had received repeated nutritional assessments prior to the start of neoadjuvant CCRT (Pre-Tx PGSGA score) and following the completion of neoadjuvant CCRT (Post-Tx PGSGA score). Accordingly, to assess the clinical significance of the PGSGA score, all patients were divided into two groups according to the PGSGA score: the low PGSGA (score **≤** 3) and high PGSGA (score > 3) groups. The high PGSGA group comprised grade B and grade C participants (the malnourished group).

### 2.3. Animals and Experimental Design

All experimental procedures involving animals were approved by the Experimental Animal Ethics Committee of Chang Gung Memorial hospital (No. 2018092510 & 2021030801). Six-week-old C57BL/6 mice were used to establish the 4-nitroquinoline 1-oxide (4NQO)-induced cancer model, as described previously [25]. The mice with 4NQO-induced esophageal tumors were divided into two groups: 4NQO-control and 4NQO-nutrition groups. For the in vivo experiments, six animals were used per group and duplicate experiments were performed. The mice were housed in a controlled environment with free access to a standard diet (5010, Labdiet) and water. The animals in the 4NQO-nutrition group were additionally given a nutrition supplement every day via oral gavage (300 µL per day) 20 weeks after the initiation of the 4NQO treatment. The nutrition supplementation for the animal study is a commercially available nutrition product (PROSURE; manufactured by Abbott (Chicago, IL, USA)), and is suitable for oncology patients. It is a 1.3 kcal/mL oral nutritional supplement enriched with protein, omega-3 fatty acids and antioxidants. Each mL of nutrition supplement contains 183 mg of carbohydrate, 66 mg of protein, 4 mg of eicosapentanenoic acid (EPA), 2 mg of docosahexaenoic acid (DHA), and multiple micronutrients. For the 4NQO-control group, mice were given normal saline via the same procedure. In vivo optical imaging was performed in 4NQO-treated mice using fluorescence molecular tomography to measure tumor formation at the indicated time prior to autopsy. The fluorescent probe 2-deoxyglucosone 750 was used for in vivo tumor imaging based on enhanced glucose uptake in tumor cells compared to surrounding nonmalignant tissues. After imaging, the presence of mouse esophageal lesions was further evaluated by gross examination of tissue samples. In addition, to determine the numbers of MDSCs, single-cell suspensions were prepared from murine spleens and then analyzed by flow cytometry gated for Gr1 and CD11b, as described previously [16,25].

### 2.4. Statistical Analysis

Clinicopathological characteristics were compared using the Student’s *t* test, chi-square test, and analysis of variance. Univariate and multivariate analyses were performed using Cox proportional hazards models. All tests were two-sided. Results of the Cox model analysis were reported with relative risks and 95% confidence intervals. The main end points were overall survival (OS), treatment response and disease status. The Kaplan–Meier method was used to calculate survival curves, and the log-rank test to determine differences between the two groups. In addition, we used the inverse probability of treatment weighting (IPTW) of the propensity scores to create a pseudo-population in which study groups were balanced across covariates, as described previously [26].

## 3. Results

### 3.1. The Nutritional Status Correlated with Tumor Progression in Esophageal Cancer Patients

**Figure 1:**
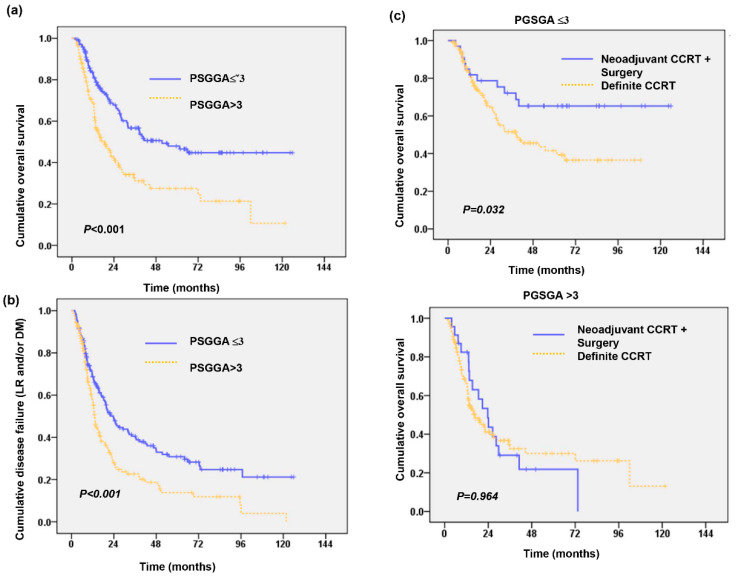
Nutrition status correlated with clinical outcome of esophageal-SCC patients. Kaplan-Meier overall survival (OS) survival curves (**a**), and cumulative disease-failure rates (**b**) of 340 patients stratified by pre-Tx PGSGA groups. Additionally, surgery significantly improved OS in patients of low pre-Tx PGSGA group but did not significantly benefit for undernourished patients (**c**).

There were 78 (23%) patients with clinical stage I–II disease and 262 (77%) with clinical stage III–IV disease. Of these patients, 284 received definitive CCRT (CCRT group), and the others received neoadjuvant CCRT followed by surgery (surgery group). The median pretreatment PGSGA score (Pre-Tx PGSGA) of the overall cohort was 3.5. There were 170 (50%) patients with a Pre-Tx PGSGA score **≤** 3 (low PGSGA group), and 170 (50%) patients with a Pre-Tx PGSGA score > 3 (high PGSGA group; malnourished group). As shown in Table 1. The high Pre-Tx PGSGA group was significantly associated with more advanced disease, lower body mass index (BMI) and a higher risk of distant metastasis and death during follow-up. We further examine whether the Pre-Tx PGSGA score was associated with the outcomes after curative treatment. As shown in Figure 1a,b, the patients in the high Pre-Tx PGSGA group had shorter OS times and higher rates of disease failure (*p* < 0.001). We further examined whether nutritional status plays a role in the treatment decision. As shown in Figure 1c, surgical resection obviously prolonged the survival time in the low Pre-Tx PGSGA group (*p* = 0.032), but not in the high PGSGA group (*p* = 0.964). Table 2 in multivariate analyses revealed that a low Pre-Tx PGSGA score, early clinical stage, response to treatment and surgery were good predictors for OS. To further corroborate the results, we used an IPTW propensity score analysis to balance the distributions of most clinicopathological characteristics between the low and high PGSGA (Table 3). The unweighted and IPTW analyses showed that low PGSGA was associated with better OS and lower risk developing distance metastasis (Table 4).

### 3.2. Relationships among the Pre-Tx PGSGA Score and the Immunologic Markers (Pre-Treatment NLR, the Levels of CD11b + CD33 + HLA–DR− Cells and IL-6)

**Figure 2:**
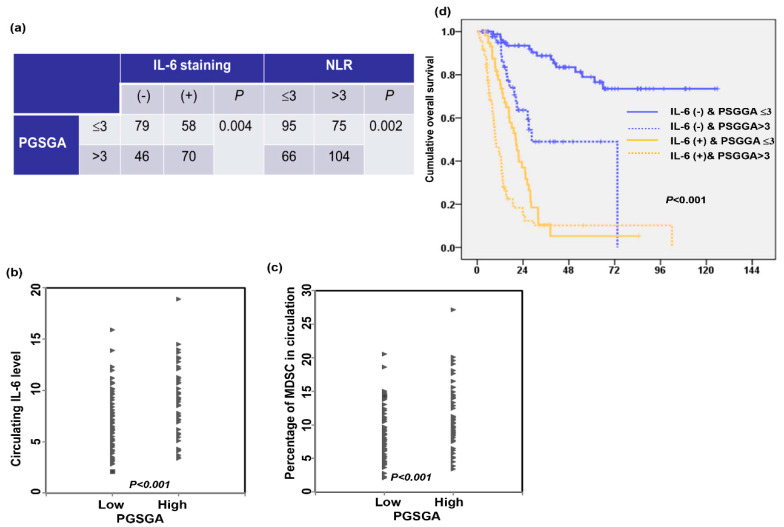
Relationships between nutrition status and immunologic markers IL-6. The nutrition status correlate with the staining of IL-6 in tumor specimens and NLR in esophageal-SCC patients (**a**). The values of circulating IL-6 (**b**) and MDSC (**c**) in the groups of esophageal-SCC patients with and without malnutrition. (**c**,**d**) The overall survival differences were according to the pre-Tx PGSGA in the group of esophageal-SCC patients with the data of IL-6 staining.

Various factors may determine immune responsiveness in cancer 18. The IL-6 and NLR, immunologically based index [14], were associated with immune evasion and nutrition metabolism in cancer. MDSCs, a population of cells with suppressive activity and measured as CD11b + CD33 + HLA–DR− cells [13], contribute to the negative regulation of immune responses that occur in cancer. We previously reported that IL-6-mediated induction of MDSCs was associated with esophageal tumor promotion and poor prognosis, and the NLR was related to the IL-6 and MDSC level. In the present study, we examined whether nutritional status was correlated with these immunologic markers in esophageal-SCC patients. Figure 2a–c shows that the Pre-Tx PGSGA score was significantly correlated to the expression levels of IL-6 in tumor specimens, and the levels of NLR, IL-6 and the percentage of MDSCs in circulation. Furthermore, by survival analyses, positive staining of IL-6 and a high Pre-Tx PGSGA score were associated with reduced OS time (Figure 2d). Based on the results, we suggest that a high Pre-Tx PGSGA score was associated with a tumor-promoting immune response, which plays a role in predicting a poor prognosis in esophageal-SCC.

### 3.3. Role of the Change in Nutritional Status during Treatment in Prognosis

Of these 340 patients, after neoadjuvant CCRT, 155 (45%) had improved nutritional status (decrease in the post-Tx PGSGA score), 97 (29%) had deteriorated nutrition status (increase in the post-Tx PGSGA score), and the other 88 patients had no change in the PGSGA score compared to pre-Tx PGSGA score (Table 5). As shown in Figure S1a, the improvement of nutritional status after neoadjuvant CCRT was significantly associated with longer survival times in malnourished patients at diagnosis. More importantly, we found that surgical resection significantly prolonged the survival time of patients in the low Pre-Tx PGSGA group and of those without deterioration in nutritional status during treatment (*p* = 0.004), but not of those in the high Pre-Tx PGSGA group or with a deteriorated PGSGA score after neoadjuvant CCRT (Figure S1b). After IPTW adjustment (Table S1), the analyses showed that low post-Tx PGSGA was associated with better OS and lower risk developing loco-regional recurrence and distance metastasis (Table 6).

### 3.4. Role of Nutrition Supplementation in Esophageal Tumor Progression and Its Relationship with the Tumor-Promoting Immune Response In Vivo

**Figure 3:**
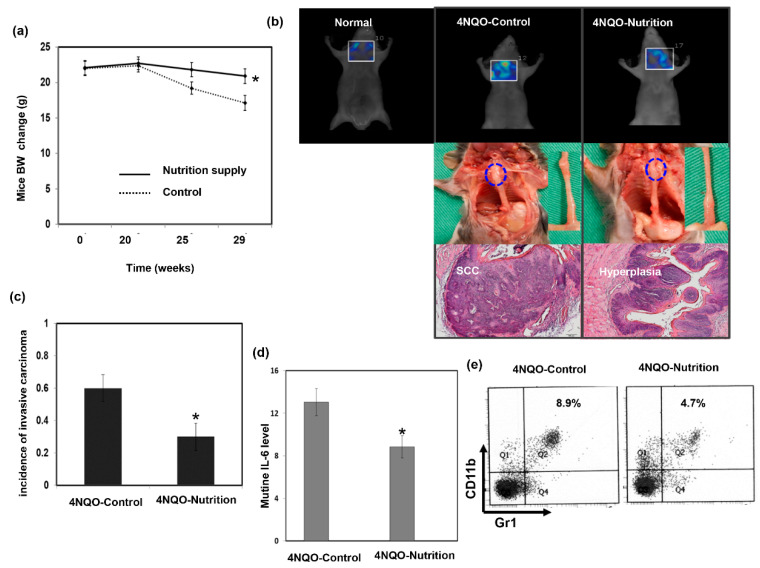
Effect of nutrition supplement on esophageal tumor progression in vivo. (**a**) The changes of body weight in animals treated with 4-nitroquinoline 1-oxide (4NQO) for 16 weeks and then followed for 12–14 weeks with or without additional nutrition supplement. Data are presented as means ± standard errors of the mean, *, *p* < 0.05. Furthermore, effects of nutrition on esophageal tumor formation in 4NQO-treated mice were determined by fluorescence molecular tomography analysis of glucose uptake combined with histology (**b**), increased incidence of developing invasive carcinoma (**c**), increased circulating IL-6 level (**d**), and flow cytometry analysis of MDSCs (**e**).

We further examined whether nutritional supplementation plays a role in tumor progression using a 4NQO-induced esophageal cancer mouse model as well as its association with immune status. Figure 3a–c shows that nutritional supplementation suppressed invasive esophageal carcinoma development associated with reduced weight loss. Moreover, animals that received oral nutrition supplementation had lower serum IL-6 levels and attenuated MDSC recruitment (Figure 3d,e). Based on the findings in vivo, we suggested that adequate nutrition supplementation and reduced body weight loss might be associated with an improvement in the immune response against cancer.

## 4. Discussion

The impairment of nutritional status occurs frequently in cancer patients, leading to worsening quality of life and even a higher mortality rate [6]. BMI and sarcopenia were used to measure nutrition status. However, nutritional problems are complicated. PGSGA is a valid and reliable nutrition assessment tool to identify malnourished cancer patients in the hospital [23]. This is a relatively large nutritional study that specifically focused on the PGSGA score in esophageal-SCC patients. There were 340 esophageal-SCC patients enrolled in this research, with ages ranging from 33 to 82 years. In the present study, there were 170 patients (50%) with a high PGSGA score (malnourished group). The incidence of malnutrition is similar to that in published research [6,7,27]. We found that advanced clinical stage was associated with increased nutritional risk before treatment. Furthermore, a high PGSGA score at diagnosis was associated with a higher risk of developing distant metastasis and reduced survival. Surgical resection is the mainstay of treatment for esophageal cancer in the past. However, the curative potential of definite CCRT has challenged the optimal treatment strategy for esophageal-SCC [28]. We examined whether surgery could bring survival benefit to esophageal-SCC patients with nutritional risk. Our data showed that surgical resection obviously prolonged the survival rate of patients with a low Pre-Tx PGSGA score but not that of malnourished patients. The patients with malnutrition were associated with a poor treatment response to neoadjuvant CCRT. Furthermore, in the surgery subgroup, the pathologic complete response (CR) rate was 57% in patients with a low PGSGA score at diagnosis, compared to 26% in malnourished patients (*p* = 0.02). Therefore, it is important to prevent malnutrition and enhance treatment efficiency and survival.

How to overcome immune suppression which promotes tumor progression and inhibits the efficiency of anticancer treatment is an important issue for cancer patients. NLR plays a key indicator in host systemic immune responses [29,30]. Studies have reported that IL-6 produced by tumors is involved in accumulation and expansion of MDSCs in tumor-bearing hosts [31]. IL-6 is suggested to modulate the local tumor microenvironment and systemic immune responses contributing to the immunosuppressive environment and allow cancer cells to acquire an advanced malignant phenotype in cancer patients [32,33]. Furthermore, IL-6 was also reported to be a key mediator in cancer-related cachexia [34,35]. Based on our previous analyses, increased IL-6 levels were significantly correlated with elevated NLR and MDSCs, which was associated with advanced clinical stage and reduced OS time. Research has been pointed out that nutrition can affect the immune response with a correlation between nutritional status and systemic inflammation in various types of cancer [6,9]. However, this correlation remains unclear in esophageal-SCC. Our data revealed that the Pre-Tx PGSGA score was significantly correlated with the level of NLR, MDSCs and the IL-6 staining in tumor specimens. Therefore, malnutrition was suggested to be a factor associated with the tumor-promoting immune response in esophageal-SCC patients.

The patients with upper gastrointestinal cancer had the risk of malnutrition induced by anticancer treatment. In the present study, 29% of patients had deteriorated nutrition status. The subgroup of patients with deteriorated nutritional status significantly correlated with poor treatment response and a high risk of developing locoregional failure and distant metastasis. We further examined whether the improvement of nutritional status contributes to better clinical outcomes. Our data demonstrated that the improvement of nutritional status and the achievement of a low Post-Tx PGSGA score significantly increased the OS time. Furthermore, the survival analysis revealed that surgical resection brought the survival benefit to patients in the low post-Tx PGSGA group, but not for the malnourished patients after neoadjuvant CCRT.

Enhancement of the patient’s immune defenses is a useful approach to reduce complications and improve prognosis. Artificial nutrition enriched in nutrients has been developed with the aim of stimulating the host immune response, improving response and increasing survival [7]. In this study, we applied oral nutritional supplements to examine the immune and tumor statuses in tumor-bearing mice. The experimental data showed that oral nutritional supplementation decreased the tumor growth associated with lower serum IL-6 levels and attenuated MDSC recruitment. Based on the findings in vivo, we suggested that adequate nutrition supplementation to reduce weight loss might be associated with an improvement in the anticancer immune response of the host.

The weakness of our study is it was a retrospective analysis of a population with different stages from a single institution. There were the potential unmeasured selection biases regarding performance status, access to healthcare, or other patient-related factors. Therefore, the issue should be further investigated by prospective study.

## 5. Conclusions

In the present study, we showed that malnutrition was related to the increased IL-6 and NLR levels and was a strong prognostic indicator for esophageal-SCC patients. We suggest nutritional assessment and appropriate nutritional supplementation can assist in making appropriate treatment decisions and improving the prognosis of esophageal-SCC patients.

## Figures and Tables

**Table 1:** Characteristics of ESCC patients correlated with the baseline nutrition status.

	No. of Patients		
	PGSGA ≤ 3	PGSSA > 3	*p* Value
**patients**	170	170	
**Age**			
Median	55.6	57.3	0.110
Range	33.8~82.8	33.7~82.6	
**Differentiation**			0.233
WD-MD	97	86	
PD	73	84	
**Clinical stage**			0.039 *
I–II	47	31	
III–IV	123	139	
**LN involvement**			0.030 *
N0-N1	91	71	
N2–N3	79	99	
**BMI**			<0.001 *
>=18	159	135	
<18	11	35	
**Tx policy**			0.145
Definite CCRT	137	147	
Surgery +/− Tx	33	23	
**Response to Neoadjuvant Tx**			0.001 *
Response	151	127	
No response	19	43	
**Loco-regional disease**			0.340
Control	88	82	
Failure	82	88	
**Distant metastasis**			0.008 *
Negative	125	102	
Positive	45	68	
**Status**			0.002 *
Alive	100	72	
Dead	70	98	

Abbreviations: Tx = treatment; CCRT = concurrent chemoradiotherapy; WD = well differentiated; MD = moderately differented; PD = poorly differentiated; BMI = body mass index; * = statistical significance.

**Table 2:** Adjusted hazard ratio of determine factors associated with OS of patients with ESCC.

Variable	HR	95% CI	*p* Value
Age			
<60	Ref		
>=60	1.07	0.78–1.48	0.68
Clinical stage			
Stage I–II	Ref		
Stage III–IV	2.31	1.50–3.57	<0.001 *
PSGGA score			
≤3	Ref		
>3	1.55	1.13–2.15	0.007 *
Treatment			
Definite CCRT	Ref		
Pre-op CCRT + surgery	0.55	0.35–0.85	0.008 *
Response to CCRT			
Response (+)	Ref		
Poor response	3.88	2.66–5.68	<0.001 *

* = statistical significance.

**Table 3:** Characteristics of patients for unweighted sample and Inverse Probability of Treatment-Weighted (IPTW-ATE) sample.

Variables	Unweighted Population, No. (%)				Standardized Difference	Weighted Population, %				Standardized Difference
	PGSGA ≤ 3		PGSGA > 3			PGSGA ≤ 3		PGSGA > 3		
**Total**	170		170							
**Age (years)**					−0.099					0.002
<60	116	(68.2)	108	(63.5)		66.3		66.3		
≥60	54	(31.8)	62	(36.5)		337		33.7		
Median (Range)	55.6	(33.8–82.8)	57.3	(33.7–82.6)	0.174	56.0	(33.8–82.8)	56.8	(33.7–82.6)	0.080
**Differentiation**					−0.130					−0.001
WD-MD	97	(57.1)	86	(50.6)		53.3		53.2		
PD	73	(42.9)	84	(49.4)		467		46.8		
**Clinical stage**					−0.225					0.001
I–II	47	(27.6)	31	(18.2)		22.8		22.9		
III–IV	123	(72.4)	139	(81.8)		77.2		77.1		
**LN involvement**					−0.237					−0.101
N0-N1	91	(53.5)	71	(41.8)		50.3		45.3		
N2–N3	79	(46.5)	99	(58.2)		49.7		54.7		
**BMI**					0.422					0.424
<18	11	(6.5)	35	(20.6)		6.4		20.5		
≥18	159	(93.5)	135	(79.4)		93.6		79.5		
**Tx policy**					0.159					−0.008
Definite CCRT	137	(80.6)	147	(86.5)		83.3		83.0		
Surgery ± Tx	33	(19.4)	23	(13.5)		16.7		17.0		
**Response to Neoadjuvant Tx**					−0.372					−0.372
Response	151	(88.8)	127	(74.7)		88.6		74.5		
No response	19	(11.2)	43	(25.3)		11.4		25.5		

**Table 4:** Odds ratios for study outcomes between low and high PG-SGA groups by different analysis approaches.

Variables	OS		LRF		Distant Metastasis	
	HR (95% CI)	*p*-Value	HR (95% CI)	*p*-Value	*p*-Value	
***Unweighted***						
**Pre-Tx PG-SGA**						
**≤3**	Reference		Reference		Reference	
>3	1.56 (1.13–2.15)	0.007	1.16 (0.84–1.60)	0.373	1.69 (1.18–2.40)	0.004
***IPTW-ATE***						
**Pre-Tx PG-SGA**						
**≤3**	Reference		Reference		Reference	
>3	1.55 (1.24–1.95)	<0.001	1.14 (0.91–1.43)	0.259	1.68 (1.31–2.14)	<0.001

**Table 5:** Characteristics of ESCC patients correlated with the post-Tx nutrition status.

	No. of Patients		
	Post-Tx_PGSGA ≤3	Post-Tx_PGSGA >3	*p* Value
**patients**	208	132	
**Pre-Tx PGSGAa**			<0.001 *
**≤3**	139	31	
>3	69	101	
**NLR**			0.003 *
**≤3**	112	49	
>3	96	83	
**BW loss**			0.008 *
<=5%	119	56	
>5%	89	76	
**Tx policy**			0.043 *
Definite CCRT	167	117	
Surgery/neoadjuvant Tx	41	15	
**Response to Neoadjuvant Tx**			<0.001 *
Response	186	92	
No response	22	40	
**Change of PGSGA**			<0.001 *
Improvement/no change	191	52	
Deterioration	17	80	
**Loco-regional disease**			0.004
Control	117	53	
Failure	91	79	
**Status**			<0.001 *
Alive	132	40	
Dead	76	92	

Abbreviations: Pre-Tx = Before neoadjuvant treatment; Post-Tx = After neoadjuvant treatment; NLR = neutrophil-to lymphocyte ratio; BW = body weight; * = statistical significance.

**Table 6:** Odds ratios for study outcomes between low and high Post-Tx PG-SGA groups by different analysis approaches.

Variables	OS		LRF		Distant Metastasis	
	HR (95% CI)	*p*-Value	HR (95% CI)	*p*-Value	*p*-Value	
**Unweighted Sample**						
**Post-Tx PG-SGA**						
**≤3**	Reference ^a^		Reference		Reference	
>3	2.59 (1.87–3.56)	<0.001	1.96 (1.41–2.73)	<0.001	1.92 (1.34–2.74)	<0.001
**IPTW-ATE**						
**Post-Tx PG-SGA**						
**≤3**	Reference		Reference		Reference	
>3	2.66 (2.12–3.32)	<0.001	1.95 (1.56–2.44)	<0.001	1.92 (1.49–2.46)	<0.001

^a^ The group of Post-Tx PG-SGA ≤ 3 is the reference group.

## Data Availability

The data presented in this study are available on reasonable request from the corresponding author.

## Supplementary Materials

The following are available online at https://www.mdpi.com/article/10.3390/nu13092997/s1, Figure S1: Role of the change in nutritional status during treatment in prognosis, Table S1: Characteristics of patients for unweighted sample and Inverse Probability of Treatment-Weighted (IPTW-ATE) sample
