# Supplementary material for: Role of Nutritional Status in the Treatment Outcome for Esophageal Squamous Cell Carcinoma
Source: Nutrients. 2021 Aug 28;13(9):2997. doi: 10.3390/nu13092997 (PMC8466664; doi:10.3390/nu13092997)

Table S1 Characteristics of patients for unweighted sample and Inverse Probability of Treatment-Weighted (IPTW-ATE) sample

Variables	Unweighted Population, No. (%)		Standardized Difference	Weighted Population, %		Standardized Difference
	Post-Tx PGSGA ≤3	Post-Tx PGSGA >3		Post-Tx PGSGA ≤3	Post-Tx PGSGA >3	
**Total**	208	132				
**Age (years)**			-0.051			0.036
<60	139 (66.8)	85 (64.4)		66.1	67.8	
≥60	69 (33.2)	47 (35.6)		33.9	32.2	
Median (Range)	55.6 (33.8-82.6)	55.6 (33.7-82.8)	0.077	55.9 (33.8-82.6)	54.3 (33.7-82.6)	-0.013
**Differentiation**			-0.256			0.013
WD-MD	121 (58.2)	62 (47.0)		53.9	54.6	
PD	87 (41.8)	70 (53.0)		46.1	46.4	
**Clinical stage**			-0.412			0.019
I-II	61 (29.3)	17 (12.9)		23	23.8	
III-IV	147 (70.7)	115 (87.1)		77	76.2	
**LN involvement**			-0.273			-0.019
N0-N1	110 (52.9)	52 (39.4)		48.2	47.3	
N2-N3	98 (47.1)	80 (60.6)		51.8	52.7	
**BMI**			0.122			0.073
<18	25 (12)	21 (15.9)		11.8	14.3	
≥18	183 (88)	111 (84.1)		88.2	85.7	
**Tx policy**			0.232			-0.048
Definite CCRT	167 (80.3)	117 (88.6)		83.3	81.6	
Surgery ± Txa	41 (19.7)	15 (11.4)		16.7	18.4	
**Response to Neoadjuvant Tx**			-0.504			-0.493
Response	186 (89.4)	92 (69.7)		89.9	70.6	
No response	22 (10.6)	40 (30.3)		10.1	29.4	

Abbreviations: a = neoadjuvant /adjuvant treatment

Figure S1 Role of the change in nutritional status during treatment in prognosis

The improvement of nutritional status during treatment increased OS time in malnourished patients(a). Additionally, surgical resection improved OS in the low Pre-Tx PGSGA group and of those without deterioration in nutritional status during treatment.

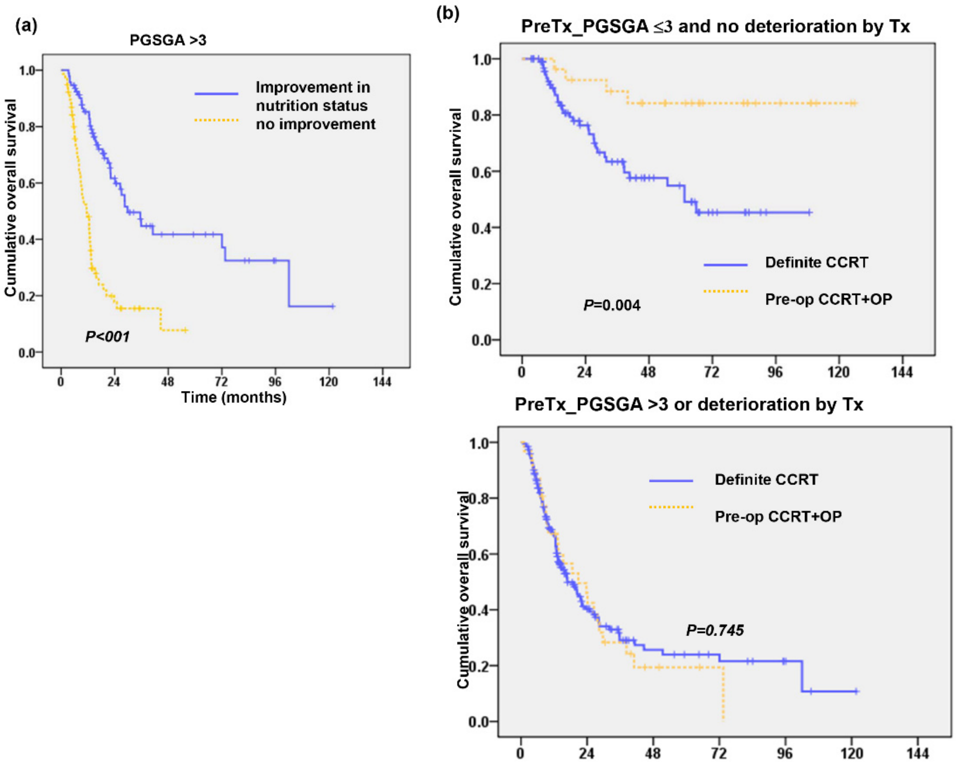

Supplement: Supplementary file 1 [file nutrients-13-02997-s001.zip › nutrients-1322402-supplementary.pdf]
